# *Dendranthema oreastrum* (Hance) Y.Ling Attenuates Oxidative Stress and Airway Inflammation in a Murine Model of Lipopolysaccharide‐Induced Acute Lung Injury

**DOI:** 10.1002/tox.24520

**Published:** 2025-04-23

**Authors:** Ji‐Hye Ha, Ba‐Wool Lee, Da‐Hye Yi, Seong‐Hun Jeong, Ju‐Hong Kim, Hyun‐Jae Jang, Ju Hwan Jeong, Ji‐Young Park, Hyung Jae Jeong, Hyung‐Jun Kwon, Young‐Bae Ryu, In‐Chul Lee

**Affiliations:** ^1^ Functional Biomaterial Research Center Korea Research Institute of Bioscience and Biotechnology Jeongeup‐si Republic of Korea; ^2^ College of Veterinary Medicine Chungnam National University Daejeon Republic of Korea; ^3^ College of Veterinary Medicine Chonnam National University Gwangju Republic of Korea; ^4^ Natural Medicine Research Center Korea Research Institute of Bioscience and Biotechnology Cheongju‐si Republic of Korea

**Keywords:** airway inflammation, *Dendranthema oreastrum* (Hance) Y.Ling, NLRP3 inflammasome, oxidative stress, TXNIP

## Abstract

We examined the antioxidant and anti‐inflammatory effects of the methanolic extract of *Dendranthema oreastrum* (Hance) Y.Ling, commonly known as “Gu‐Jeol‐Cho” in Korea and widely found across East Asia, using an LPS‐induced acute lung injury (ALI) mouse model and TNF‐α‐stimulated NCI‐H292 cells. LPS was intratracheally administered at 20 μg/50 μL/mouse for 3 days. From test day 1 to 5, experimental mice were administered DO (100 and 200 mg/kg/day) or dexamethasone (3 mg/kg/day). DO treatment decreased pro‐inflammatory cytokines in bronchoalveolar lavage fluid and TNF‐α‐stimulated NCI‐H292 cells. Additionally, DO treatment decreased alveolar wall thickening and inflammatory cell infiltration in lung tissues. DO down‐regulated TXNIP expression, inhibiting NF‐κB activation. Furthermore, DO administration reduced NLRP3 inflammasome activity by preventing the activation of caspase‐1 and IL‐1β. Additionally, DO promoted the nuclear translocation of Nrf2‐related factors, leading to an upregulation of antioxidant enzymes. The study demonstrated that DO administration markedly decreased reactive oxygen species and lipid peroxidation. These observations indicate that DO is a therapeutic agent for ALI.

## 1 Introduction

Acute lung injury (ALI) is a severe inflammatory condition of the lungs, marked by interstitial edema, damage to alveolar epithelial cells, and the accumulation of neutrophils in the lung tissue [1, 2]. It induces a high morbidity and mortality rate, with the latter estimated to be approximately 35%–40% [2]. The most common causes of ALI are pneumonia, aspiration, and sepsis due to bacterial infections [3]. Currently, there are no effective pharmacological treatments available for ALI [4]. Thus, it is crucial to identify potential therapeutic agents and develop novel treatment strategies for ALI.

The intratracheal introduction of lipopolysaccharide (LPS), a key component of the outer membrane of gram‐negative bacteria, stimulates the production of reactive oxygen species (ROS) and activates NF‐κB, a crucial regulator of immune and inflammation [5]. Once activated, the p65 NF‐κB dimer moves into the nucleus, where it binds to DNA and promotes the expression of inflammatory mediators such as tumor necrosis factor (TNF)‐α and interleukin (IL)‐1β, in an LPS‐induced ALI model [6, 7]. Additionally, LPS enhances NF‐κB binding to the NLRP3 promoter, leading to the activation of the NF‐κB‐dependent NLRP3 inflammasome and facilitating the conversion of pro‐IL‐1β into its active form in an LPS‐induced ALI mouse model [7, 8, 9, 10].

Thioredoxin‐interacting protein (TXNIP) and thioredoxin (Trx) play essential roles in maintaining cellular redox homeostasis, regulating inflammation, and contributing to the progression of various diseases, including autoimmune disorders, pulmonary fibrosis, and ALI [11, 12]. Under oxidative stress conditions, TXNIP relocates to the cytoplasm or mitochondria, where it binds to Trx, suppressing its antioxidative activity [10]. Furthermore, TXNIP is a key link between inflammation and oxidative stress in the LPS‐induced ALI model [13]. In addition to NF‐κB, TXNIP directly interacts with NLRP3, promoting the activation of the NLRP3 inflammasome [11]. Studies have shown that inhibiting the p65NF‐κB and TXNIP/NLRP3 pathways plays a crucial role in controlling LPS‐induced ALI [13]. In addition, recent research indicates that inhibiting the TXNIP/NLRP3 inflammasome can mitigate LPS‐induced ALI, suggesting that the NLRP3 inflammasome is a potential therapeutic target for inflammatory diseases [11, 14, 15, 16, 17].

Research has shown that LPS enhances inflammatory responses and oxidative stress in an experiment ALI model [18]. The transcription factor Nrf2, which is closely associated with inflammation and oxidative stress, has been widely studied for its involvement in various diseases, including degenerative conditions and ALI [19, 20, 21]. Nrf2 dissociates from Kelch‐like ECH‐associated protein 1, translocates into the nucleus, and interacts with antioxidant response elements in the promoter regions of cytoprotective genes such as heme oxygenase‐1 (HO‐1) and NAD(P)H quinone dehydrogenase‐1 (NQO‐1) [20, 21, 22, 23]. Activation of the Nrf2/HO‐1/NQO‐1 pathway has been reported to mitigate inflammatory and oxidative stress in a murine model of ALI induced by LPS [24, 25, 26]. Consequently, numerous studies have explored the potential protective effects of natural compounds with anti‐oxidant and anti‐inflammatory properties against ALI [27, 28].

*Dendranthema oreastrum* (Hance) Y.Ling, an autumn‐blooming plant native to East Asia, has been traditionally utilized in Korean medicine. Its flowers are brewed as tea and valued for treating gastroenteric disorders, bronchitis, cough, and pneumonia [29]. Previous research has indicated that DO extract exhibits strong antioxidant and anti‐inflammatory effects in LPS‐induced RAW264.7 cells [30]. Furthermore, there is currently no empirical data to support the antioxidant and anti‐inflammatory properties of DO in ALI. Consequently, we conducted an evaluation of the effects of the methanol extract of DO on the LPS‐induced ALI mouse model and the TNF‐α‐induced NCI‐H292 cells.

## 2 Materials and Methods

### 2.1 UPLC Q‐ToF/MS Analysis

The methanolic extract of *Dendranthema oreastrum* (Hance) Y.Ling was obtained from Chungcheongnam‐do, Republic of Korea, via the Korea Research Institute of Bioscience and Biotechnology (KRIBB) Extract Bank (PB4888.1). Compound identification in the aerial parts was conducted using UPLC Q‐ToF mass (Waters Corp, Milford, MA, USA) with a BEH C18 column, employing leucine‐enkephalin as the lock mass ([M + H]^+^ m/z 556.2771). Data analysis was performed using UNIFI software (Waters Corporation).

### 2.2 Cell Culture and Cell Viability Assay

NCI‐H292 human airway epithelial cells (ATCC; Rockyville, MD, USA) were cultured in Roswell Park Memorial Institute (RPMI) 1640 media (Gibco, San Diego, CA, USA) supplemented with fetal bovine serum (FBS; Gibco) and antibiotics (37°C, 5% CO_2_). To evaluate cell viability following treatment with DO and linarin, an MTT assay (3‐(4,5‐dimethylthiazol‐2‐yl)‐2,5‐diphenyltetrazolium bromide) (Amersco, SF, USA) was performed. Cells (5 × 10^4^ cells/well) were seeded in 96‐well plates and incubated for 24 h before being treated to concentrations of DO (0, 25, 50, 100, and 150 μg/mL) and linarin (0, 12.5, 25, 50, 100, and 200 μg/mL). Cytotoxicity was determined by measuring absorbance at 570 nm with a microplate reader (iMark, Bio‐Rad Laboratories, Richmond, CA, USA).

### 2.3 Measurement of Inflammatory Cytokines in TNF‐α‐Stimulated NCI‐H292 Cells

The cells were treated with DO at 0, 50, 100, and 150 μg/mL and Dexamethasone (DEX; 20 μg/mL) for 1 h before treatment with 30 ng/mL human recombinant TNF‐α. The levels of TNF‐α, IL‐6, and IL‐1β in culture medium were determined at 24 h incubation, after TNF‐α treatment and quantified using a competitive ELISA kit (RayBiotech Inc., Norcross, GA, USA) according to the manufacturer's instructions. Absorbance was measured at 450 nm using a microplate reader (Bio‐Rad Laboratories). Following the same procedure, the supernatants were collected, and the levels of TNF‐α, and IL‐6 in the culture media were quantified using ELISA kit (BD Biosciences, San Jose, CA, USA) according to manufacturer's instructions (Supporting Information Figure 1B,C). The total RNeasy kit (Qiagen, Valencia, CA, USA), 1 μg of RNA was used to synthesize single‐stand cDNA. Real‐time PCR was performed in tri‐plicate using the CFX96 Touch system (Bio‐Rad Laboratories) following the manufacturer's instructions. The PCR reaction was prepared with the SensiFAST SYBR No‐ROX kit (BioLine, Tauton, MA, USA). The primer sequences were used: TNF‐α forward, DNA‐CT TCT CAT TCC TGC TTG TGG C′, TNF‐α reverse, DNA‐GA GGG AGG CCA TTT GGG AAC′; IL‐6 forward, DNA‐AT GAT GGA TGC TAC CAA ACT GGA′, IL‐6 reverse, DNA‐TC TGA AGG ACT CTG GCT TTG TC′; IL‐1β forward, DNA‐TT CCT GAA CTC AAC TGT GAA ATG C′, IL‐1β reverse, DNA‐TT GAT GTG CTG CGA GA′ according to the manufacturer's instructions (Supporting Information Figure 1D,F).

### 2.4 Measurement of Oxidative Stress Marker and ROS Production in TNF‐α‐Stimulated NCI‐H292 Cells

Oxidative stress markers were measured as previously described [31]. The 2′,7′‐dichlorofluorescein diacetate (DCF‐DA) cellular ROS detection assay kit (Thermo Scientific, Waltham, MA, USA), thiobarbituric acid‐reactive substances (TBARS), and reduced glutathione (GSH) activity assay kit (DoGen, Republic of Korea) were used according to the manufacturer's instructions.

### 2.5 Immunoblotting

Western blot analysis was conducted following a previously described protocol [31]. The primary antibodies used in this study were as follows: p65NF‐κB, p‐p65NF‐κB, caspase‐1, IL‐1β, HO‐1, NQO‐1, and Nrf‐2 (1:1000 dilution, Abcam, Cambridge, UK), TXNIP (1:1000 dilution, Novus Bio, Centennial, CO, USA), NLRP3, β‐actin (1:1000 dilution, Cell Signaling Technology, Danvers, MA, USA), and α‐tubulin, Lamin B (1:1000 dilution, Thermo Scientific). Protein band intensities were quantified using a chemiluminescent scanner (LI‐COR, Biosciences, Lincoln, NE, USA).

### 2.6 Animal Experimental Procedure

The experimental procedures for the LPS‐induced ALI mouse model were reviewed and approved by the animal ethics committee of KRIBB (permit number: KRIBB‐AEC‐18205). Animal care and housing were conducted in accordance with previously established guidelines [32]. Mice were randomly assigned to five groups (*n* = 7/group) as follows: normal control (NC) group, LPS‐treated group (LPS, 20 μg/50 μL on day 3), dexamethasone‐treated group (DEX, 3 mg/kg from day 1 to day 5), and DO treatment groups (DO 100 and 200 mg/kg from day 1 to day 5). DEX (3 mg/kg/day) was used as a positive control and administered orally from test day 1 to day 5 [31]. DO dose was selected based on previous studies [30]. Following the final treatment, mice were anesthetized, and bronchoalveolar lavage fluid (BALF) was collected as described previously [31]. Inflammatory cells in BALF were stained using the Diff‐Quik staining reagent for further analysis.

### 2.7 Measurement of Inflammatory Cytokine Levels in BALF and Lung Tissue From LPS‐Induced ALI Model

The levels of TNF‐α, IL‐6, and IL‐1β in BALF and lung tissue were assessed using ELISA kits (R&D system, Minneapolis, MN, USA), following the manufacturer's instructions. Absorbance was detected with a microplate reader (iMark, Bio‐Rad Laboratories).

### 2.8 Histopathology of Lung Tissue From LPS‐Induced ALI Model

Lung tissues were fixed in a suitable fixative, embedded in paraffin, and sectioned into 4 μm‐thick slices. The sections were then stained with hematoxylin and eosin (H&E; BBC Biochemical, Mount Vemon, WA) to evaluate histopathological changes. Leukocyte infiltration and thickening of the alveolar wall were scored. Quantitative analysis was conducted using a Leica Microsystems (×10 objective) in a blind manner.

### 2.9 Oxidative Stress Markers Analysis in Lungs From LPS‐Induced ALI Model

Lung tissue samples were collected and processed following previously established protocols [31]. To assess oxidative stress, the supernatant obtained from processed lung tissues was analyzed for GSH (EZ‐GSH assay kit, DoGen) and TBARS levels (EZ‐TBARS assay kit, DoGen). Absorbance was measured at 415 and 540 nm using a microplate reader.

### 2.10 Statistical Analysis

The data were presented as the mean ± SD and were analyzed using one‐way ANOVA (GraphPad Software, CA, USA). A *p*‐value ≤ 0.05 were considered to have statistically significant.

## 3 Results

### 3.1 Tentative Characterization of the DO

**FIGURE 1:**
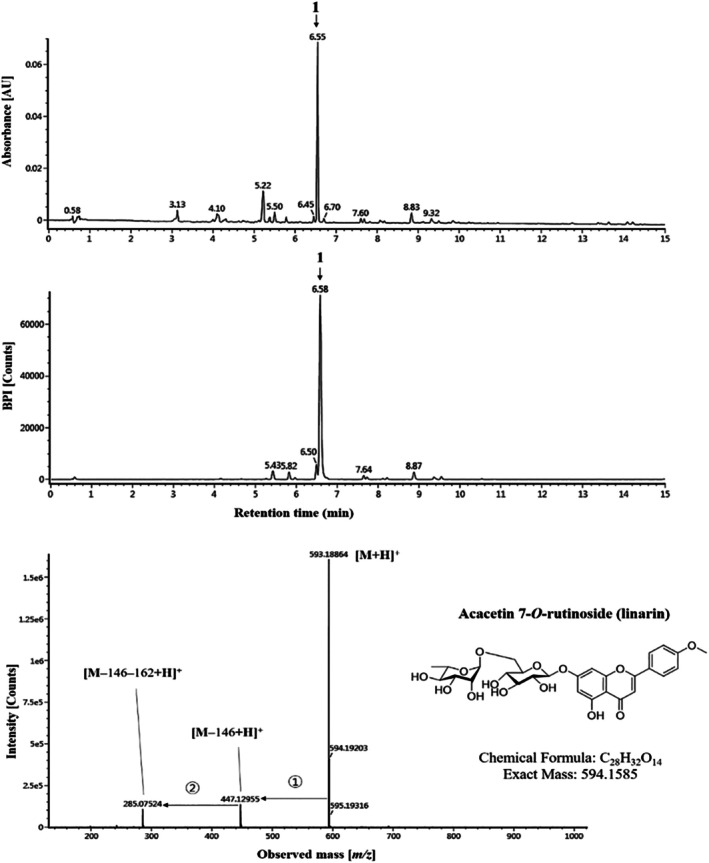
Tentative characterization of DO. The graphs show the major peaks detected by mass spectrometric analysis. Based on the fragmentation pattern in the mass spectra, peak 1 was identified as acacetin 7‐*O*‐rutinoside (linarin; C_28_H_32_O_14_).

Major peak was observed in the methanolic extract of *Dendranthema oreastrum* (Hance) Y.Ling and was analyzed using UPLC Q‐TOF/MS (Table 1 and Figure 1). The compounds in DO were tentatively identified by comparison of their accurate mass and fragmentation patterns with previously reported data. Acacetin 7‐*O*‐rutinoside (linarin; C_28_H_32_O_14_) has a molecular weight of 594, with a primary peak at m/z 655 [M+H]^+^ producing characteristic ions m/z 447 and 285 due to the loss of a rhamnoside residue (146 Da) and a glucoside residue (162 Da), respectively. As 7‐*O*‐rutinoside is a key derivative in *Dendranthema oreastrum* species [33, 34] major peak 1 was tentatively identified as acacetin 7‐*O*‐rutinoside based on its fragmentation pattern and previous reports [33, 34, 35, 36].

**TABLE 1:** Tentative identification of major peak detected in DO.

No.	RT (min)	Formula	Calculated m/z	Observed m/z	Error (ppm)	Tentative compound	Fragments	References
1	6.55	C_28_H_32_O_14_	593.18648	594.18864	3.64	Acacetin 7‐O‐rutinoside	447, 285, 270	[21, 29, 33]

### 3.2 Effects of DO and Linarin on Pro‐Inflammatory Cytokines in TNF‐α‐Stimulated NCI‐H292 Cells

**FIGURE 2:**
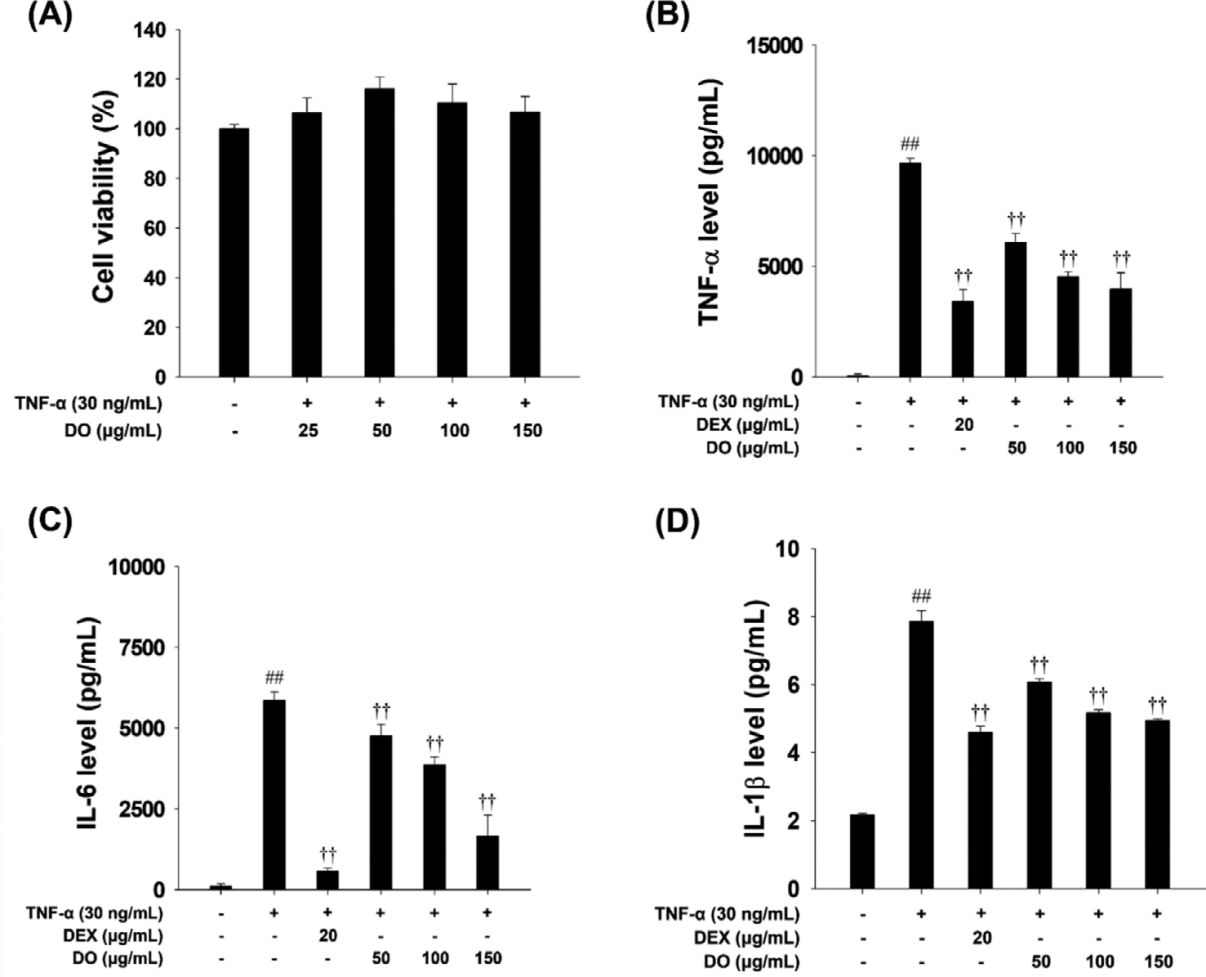
Effect of DO on inflammatory responses in NCI‐H292 cells. (A) Cell viability was assessed using the MTT assay following treatment with DO (0, 25, 50, 100, and 150 μg/mL) for 24 h. The concentrations of (B) TNF‐α, (C) IL‐6, and (D) IL‐1β in TNF‐α‐stimulated NCI‐H292 cells were quantified by ELISA. The values are expressed as the means ± SD (*n* = 3). Significance: ^##^ *p* < 0.01 vs. control; ^††^ *p* < 0.01 vs. TNF‐α‐stimulated cells, respectively.

DO treatment exhibited no cytotoxic effect on NCI‐H292 cells at concentrations 0, 50, 100, and 150 μg/mL (Figure 2A). TNF‐α‐treated cells led to a significant increase in the levels of pro‐inflammatory cytokines, including TNF‐α, IL‐6, and IL‐1β compared to non‐stimulated cells. However, TNF‐α‐stimulated pro‐inflammatory cytokines were dose‐dependently decreased by treatment DO (Figure 2B–D). Similarly, linarin was treated at nontoxic concentrations (0, 12.5, 25, 50, 100, and 200 μg/mL), as determined in Supporting Information Figure 1A. TNF‐α stimulation resulted in a noticeable increase in the production of TNF‐α and IL‐6 compared to the control (Supporting Information Figure 1B,C). However, linarin treatment significantly suppressed TNF‐α, IL‐6, and IL‐1β expression levels compared to TNF‐α‐treated cells (Supporting Information Figure 1D,F).

### 3.3 Effects of DO and Linarin on p65NF‐κB and TXNIP/NLRP3 Inflammasome Pathway in TNF‐α‐Stimulated NCI‐H292 Cells

**FIGURE 3:**
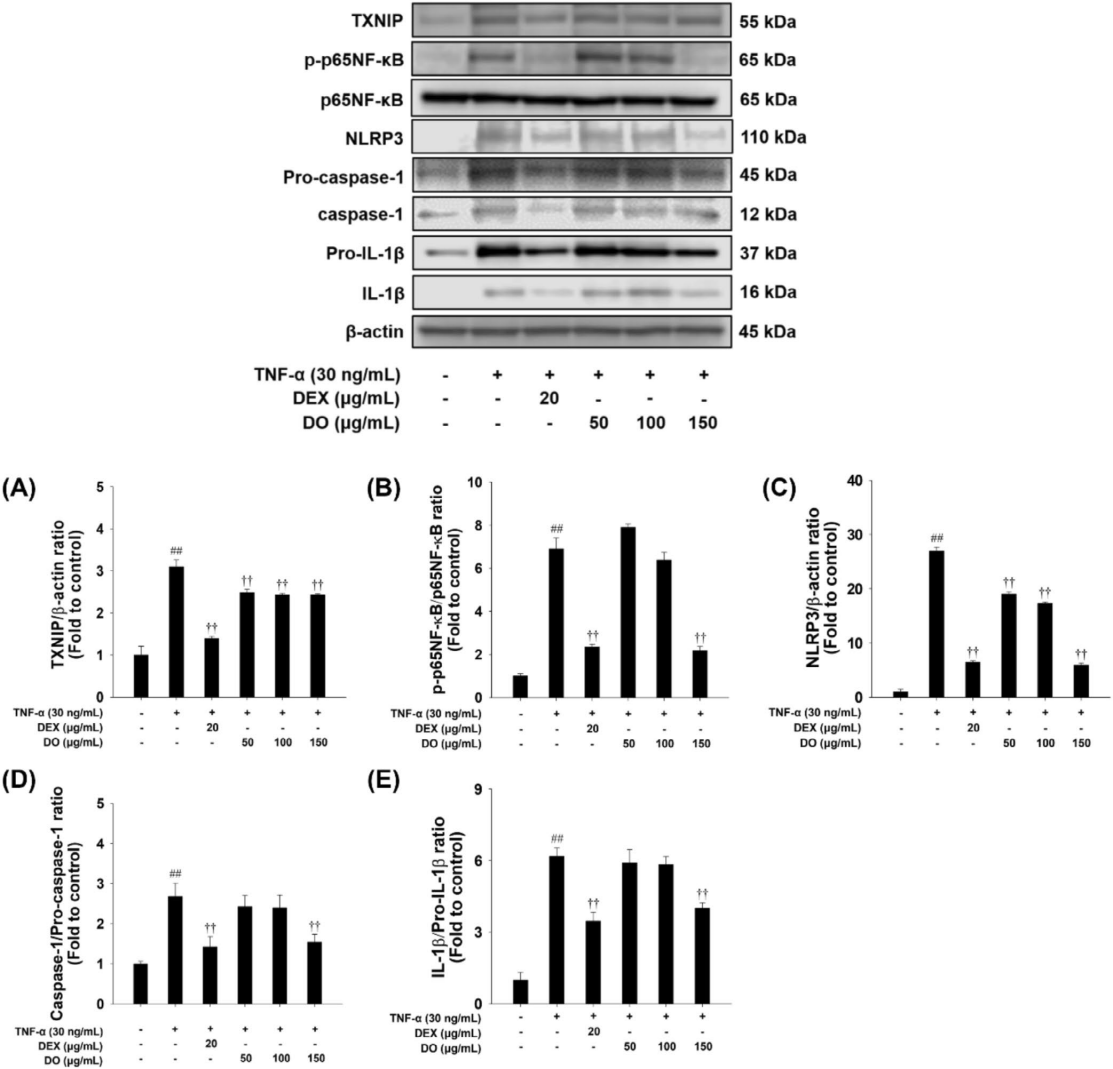
Effects of DO on the activation of the p65NF‐κB and TXNIP/NLRP3 inflammasome pathway in NCI‐H292 cells. The expression of (A) TXNIP, (B) p65NF‐κB, (C) NLRP3, (D) caspase‐1, and (E) IL‐1β. The values are expressed as the means ± SD (*n* = 3). Significance: ^##^ *p* < 0.01 vs. control; ^††^ *p* < 0.01 vs. TNF‐α‐stimulated cells, respectively.

TNF‐α‐treated cells showed a marked phosphorylation of treatment and markedly increased the upregulation of NLRP3 with an increase in the activated forms of caspase‐1 and IL‐1β in NCI‐H292 cells. However, DO treatment effectively inhibited the phosphorylation of p65NF‐κB and suppressed TXNIP expression compared to TNF‐α‐stimulated cells (Figure 3A,B). Additionally, DO treatment resulted in a dose‐dependent reduction in NLRP3 expression as well as the activated forms of caspase‐1 and IL‐1β (Figure 3C,D). Similarly, linarin treatment decreased the TNF‐α‐treated upregulation of TXNIP and p65NF‐κB expression (Supporting Information Figure 2A,B). Moreover, TNF‐α‐treated cells exhibited significantly elevated NLRP3, the activated forms of caspase‐1 and IL‐1β expression compared to untreated cells. In contrast, linarin‐treated cells showed a noticeable decrease in the expression of NLRP3, caspase‐1, and IL‐1β compared to TNF‐α‐stimulated cells (Supporting Information Figure 2C,E).

### 3.4 Effects of DO and Linarin on Oxidative Stress Markers, ROS Production, and Nrf‐2/NQO‐1/HO‐1 Pathway in TNF‐α‐Stimulated NCI‐H292 Cells

**FIGURE 4:**
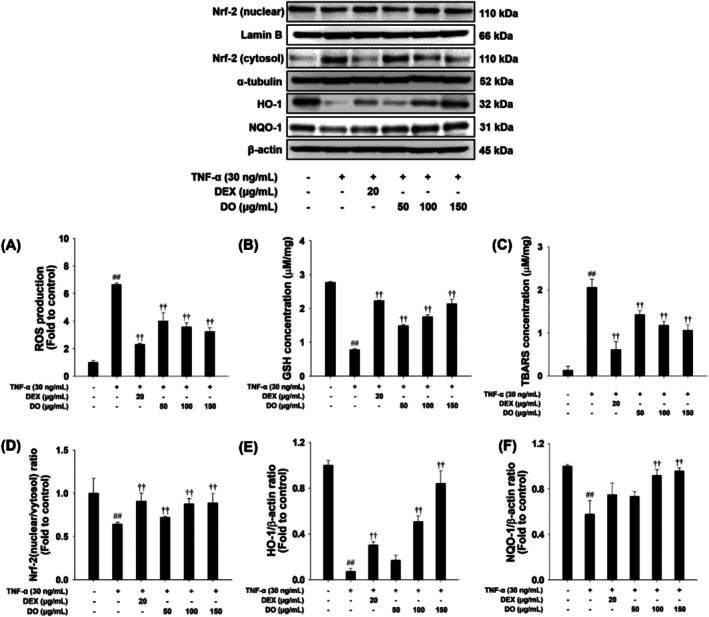
Effects of DO on ROS production, oxidative stress, and Nrf‐2 pathway in NCI‐H292 cells. The expression of (A) ROS production, (B) GSH, and (C) TBARS. The expression of (D) Nrf‐2, (E) HO‐1, and (F) NQO‐1. The values are expressed as the means ± SD (*n* = 3). Significance: ^##^ *p* < 0.01 vs. control; ^††^ *p* < 0.01 vs. TNF‐α‐stimulated cells, respectively.

TNF‐α treated cells increased ROS and TBARS levels and decreased GSH content in NCI‐H292 cells (Figure 4). Additionally, TNF‐α treatment suppressed the nuclear translocation of Nrf‐2, concurrent with the reduction of NQO‐1 and HO‐1 expression in NCI‐H292 cells compared to the unstimulated cells. In contrast, DO treatment effectively reduced ROS and TBARS levels while restoring GSH content in TNF‐α stimulated NCI‐H292 cells (Figure 4A–C). Moreover, DO treatment promoted Nrf‐2 activity and significantly upregulated the expression levels of NQO‐1 and HO‐1 (Figure 4D–F). Similarly, linarin treatment reduced ROS and TBARS levels while increasing GSH content in NCI‐H292 cells (Supporting Information Figure 3A–C). Furthermore, linarin‐treated cells exhibited pronounced Nrf‐2 activity, accompanied by elevated expression levels of NQO‐1 and HO‐1 (Supporting Information Figure 3D–F).

### 3.5 Effects of DO on Inflammatory Cell Counts and Pro‐Inflammatory Cytokine in BALF and Lung Tissue

**FIGURE 5:**
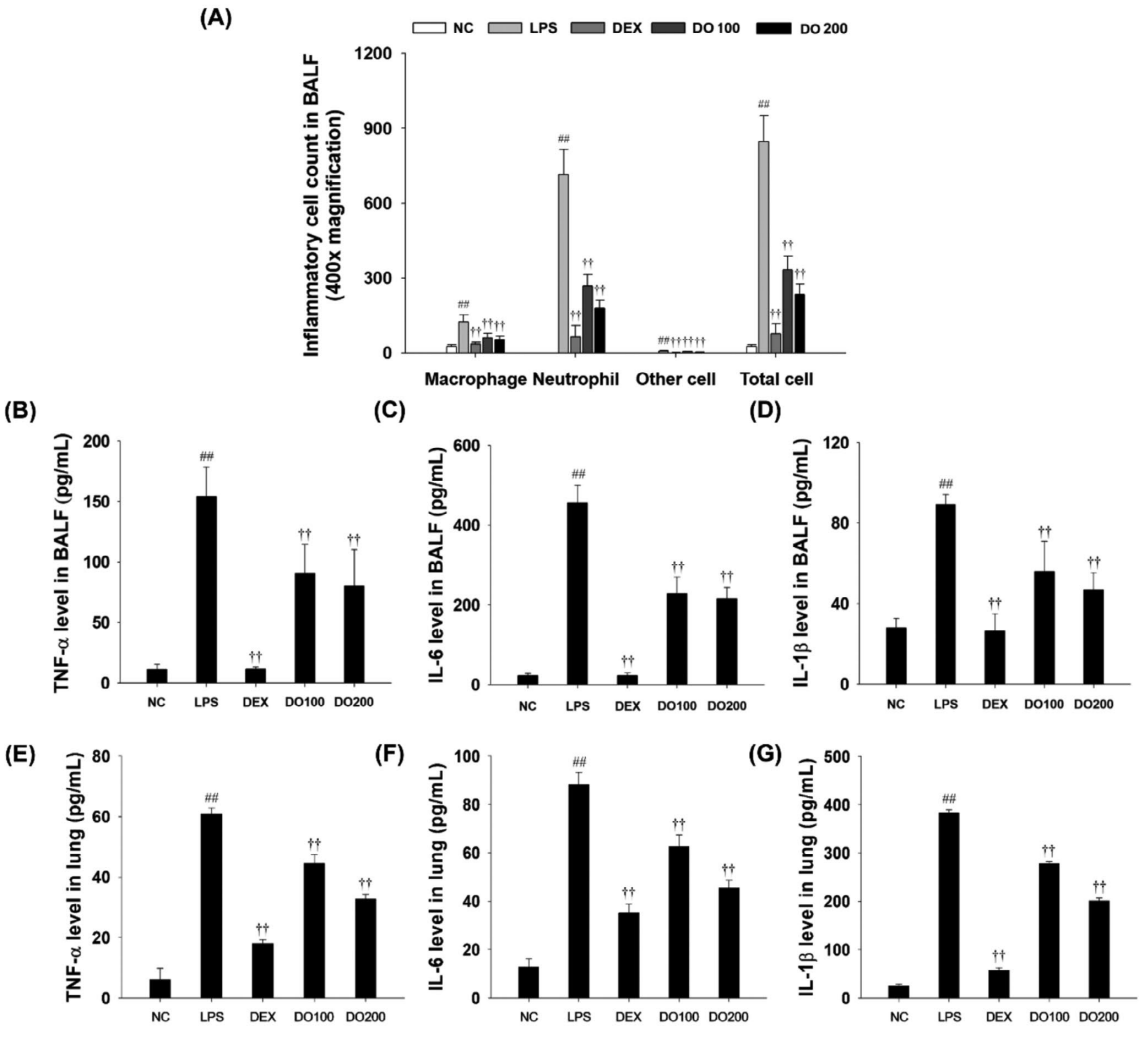
Effects of DO on pathophysiological alterations in BALF and lung tissues. (A) Differential inflammatory cells were counted by Diff‐Quick stain reagent and counted in a double‐blind manner on 3 areas for each slide. (B) TNF‐α, (C) IL‐6, and (D) IL‐1β production in BALF. The levels of (E) TNF‐α, (F) IL‐6, and (G) IL‐1β in lung tissue. The values are expressed as the means ± SD (*n* = 7). Significance: ^##^ *p* < 0.01 vs. NC; ^††^ *p* < 0.01 vs. LPS, respectively.

To evaluate the effects of DO on inflammatory cells in BALF, the counts of neutrophils, macrophages, and other immune cells were analyzed. As shown in Figure 5, LPS‐treated mice showed a significant increase in neutrophils, macrophages, and other inflammatory cells in BALF compared to the NC mouse. Additionally, pro‐inflammatory cytokine levels (TNF‐α, IL‐6, and IL‐1β) were markedly elevated in the LPS‐treated group compared with those in the control group. However, DO treatment resulted in a dose‐dependent reduction of inflammatory cells, particularly neutrophils and macrophages, in the BALF and pro‐inflammatory cytokine levels (TNF‐α, IL‐6, and IL‐1β) compared to the LPS‐treated group (Figure 5B–D). Similarly, cytokine levels in the lung tissues of the LPS‐treated group were also reduced in a dose‐dependent manner with DO treatment (Figure 5E–G).

### 3.6 Effects of DO on LPS‐Induced Lung Histological Changes in Lung Tissue

**FIGURE 6:**
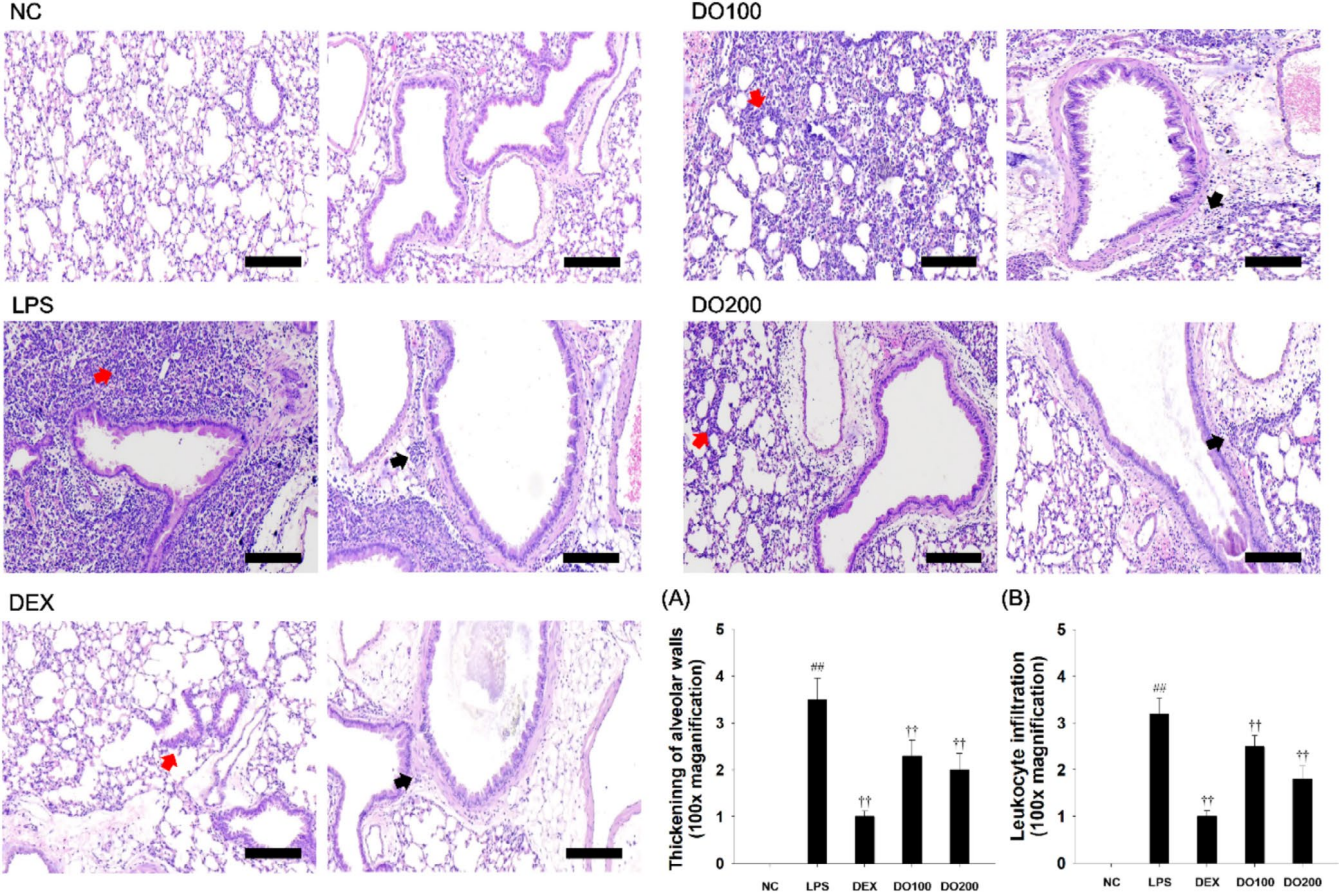
Effects of DO on airway inflammation in the lungs of the ALI model. Histological examination of airway inflammation performed in the lung tissue by H&E staining (red arrows: Leukocyte infiltration; black arrows: Alveolar wall thickening). Scale bars = 200 μm. (A) Thickening of alveolar walls and (B) inflammatory cell infiltration is expressed as the means ± SD (*n* = 7). Significance: ^##^ *p* < 0.01 vs. NC; ^††^ *p* < 0.01 vs. LPS, respectively.

The effects of DO on LPS‐induced histopathological changes in lung tissue were evaluated (Figure 6). H&E‐stained lung sections from LPS‐treated mice showed alveolar wall thickening and inflammatory cell infiltration in peribronchiolar and perivascular lesions compared to the NC. In contrast, DO pretreatment alleviated alveolar wall thickening and decreased inflammatory cell infiltration, leading to a significant decrease in lesion scores compared to the LPS‐induced mice (Figure 6A,B).

### 3.7 Effects of DO on p65NF‐κB and TXNIP/NLRP3 Inflammasome Pathways in Lung Tissue

**FIGURE 7:**
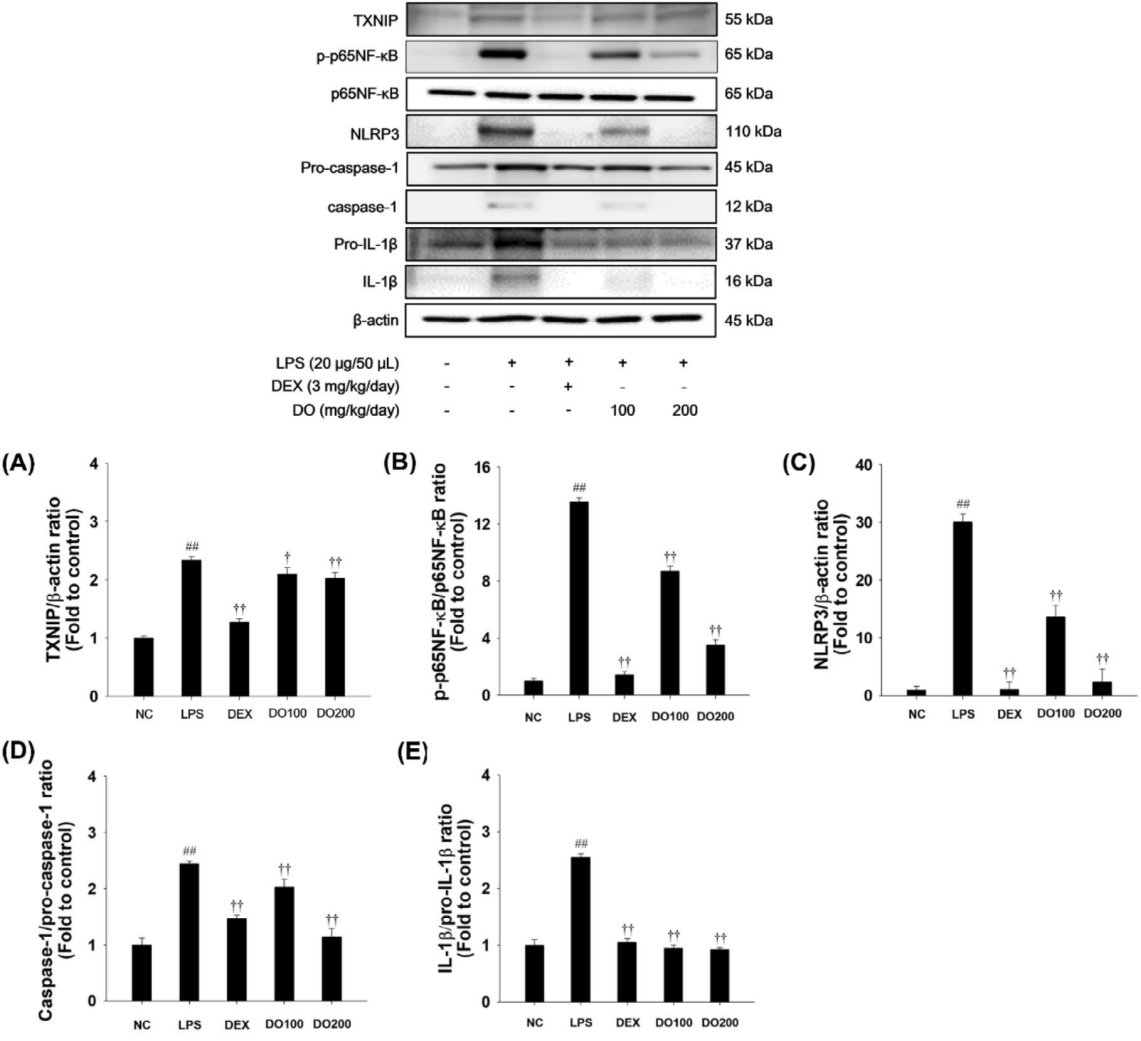
Effects of DO on activation of p65NF‐κB and TXNIP/NLRP3 inflammasome pathway in ALI model. The expression of (A) TXNIP, (B) p65NF‐κB, (C) NLRP3, (D) caspase‐1, and (E) IL‐1β. The values are expressed as the means ± SD (*n* = 7). Significance: ^##^ *p* < 0.01 vs. NC; ^†,††^ *p* < 0.05 and < 0.01 vs. LPS, respectively.

To investigate the underlying mechanism of DO treatment in attenuating LPS‐induced ALI in mice, we investigated the effects of DO on the p65NF‐κB and TXNIP/NLRP3 inflammasome in the lung tissues. As shown in Figure 7, LPS‐treated mice demonstrated a marked elevation in the phosphorylated form of p65NF‐κB and upregulation of TXNIP in lung tissues when compared with NCs. In addition, LPS instillation caused upregulation of NLRP3 with elevated activated forms of caspase‐1 and IL‐1β in lung tissues compared with the NCs. In contrast, DO treatment markedly inhibited the phosphorylation of p65NF‐κB and reduced TXNIP levels in lung tissues compared to the LPS‐treated mice (Figure 7A,B). DO treatment notably reduced the NLRP3 levels and the active forms of caspase‐1 and IL‐1β in LPS‐treated mice (Figure 7C–E).

### 3.8 Effects of DO on Oxidative Stress Markers and Nrf‐2 Pathway in Lung Tissue

**FIGURE 8:**
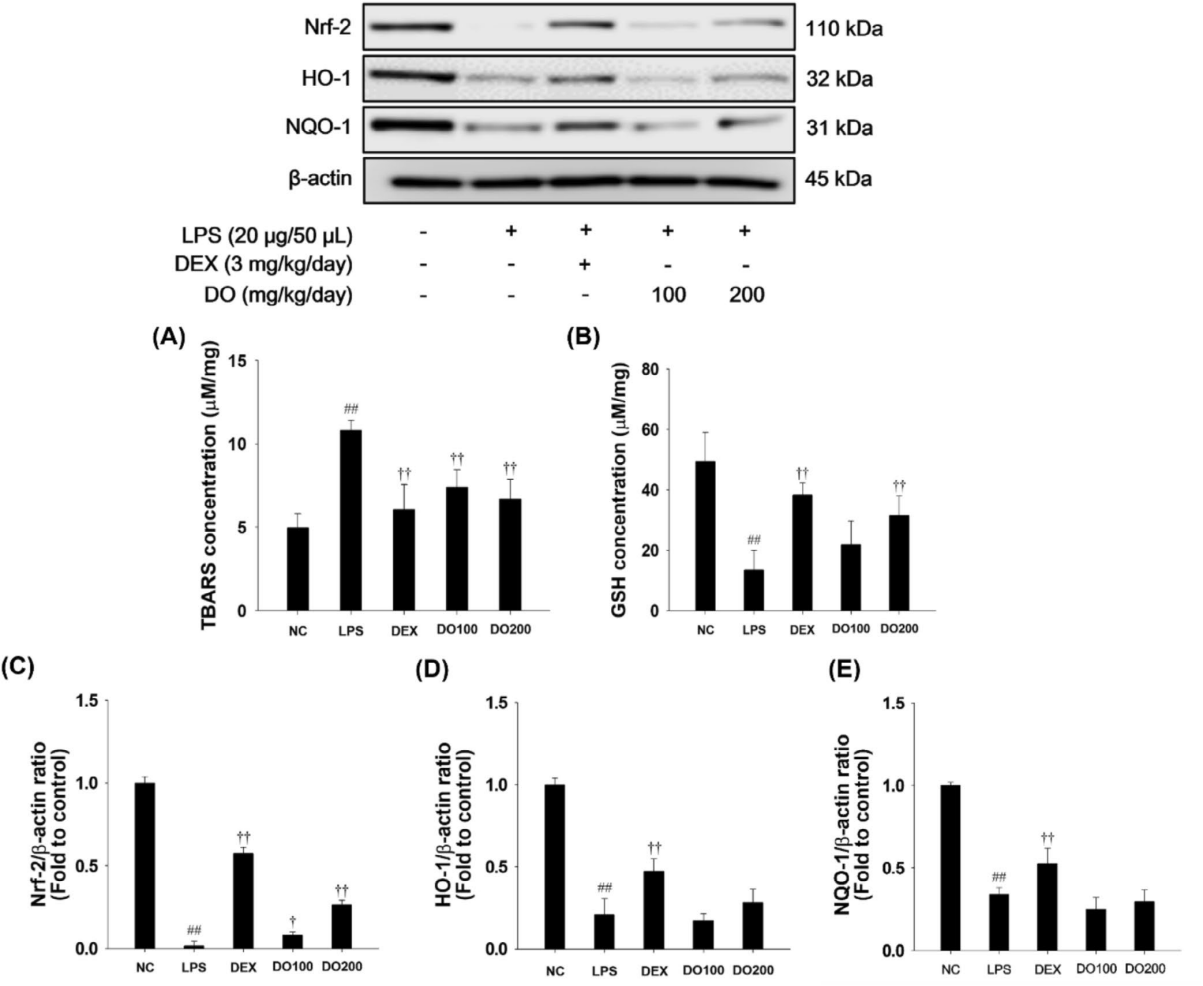
Effects of DO on oxidative stress and Nrf‐2 pathway in ALI model. The levels of (A) TBARS and (B) GSH were measured in lung tissue. The expression of (C) Nrf‐2, (D) HO‐1, and (E) NQO‐1 was assessed. The values are expressed as the means ± SD (*n* = 7). Significance: ^##^ *p* < 0.01 vs. NC; ^†,††^ *p* < 0.05 and < 0.01 vs. LPS, respectively.

As shown in Figure 8, LPS treatment led to an increase in TBARS levels and a reduction in GSH content compared to the NCs. LPS‐treated mice exhibited a significant decrease in nuclear Nrf‐2 with suppression of HO‐1 and NQO‐1 expression in lung tissues. Conversely, administration resulted in a decrease in TBARS and an increase in GSH content in lung tissues compared to LPS‐induced mice (Figure 8A,B). Moreover, DO treatment promoted Nrf‐2 activity, accompanied by elevated HO‐1 and NQO‐1 expression in lung tissues compared with LPS‐treated mice (Figure 8C,D).

## 4 Discussion

This study examined the effects of the DO methanolic extract on airway inflammation and oxidative stress in TNF‐α‐stimulated NCI‐H292 cells and LPS‐induced ALI mouse model. The administration leads to the suppression of p65NF‐κB and TXNIP/NLRP3 inflammasome pathways activation, accompanied by a significant decrease in pro‐inflammatory cytokine levels, including TNF‐α, IL‐1β, and IL‐6. DO treatment upregulated Nrf‐2 expression, which resulted in the elevation of antioxidant enzymes, such as NQO‐1, HO‐1, and GSH, and attenuated ROS generation and lipid peroxidation.

LPS triggers lung inflammation in ALI by promoting neutrophil accumulation and compromising the endothelial/epithelial barrier [35]. This activates macrophages and neutrophils, resulting in the secretion of pro‐inflammatory cytokines, which further aggravate lung injury [21, 36]. Furthermore, DO treatment significantly decreased the concentrations of TNF‐α, IL‐6, and IL‐1β in the BALF and NCI‐H292 cells. Correspondingly, it significantly reduced inflammatory cell infiltration, particularly neutrophils and macrophages, in BALF while alleviating alveolar wall thickening and inflammatory cell accumulation in lung tissue. In addition, further studies indicate that linarin, the major active compound of DO, could play a role in inhibiting inflammatory cytokine responses in TNF‐α‐stimulated NCI‐H292 cells. Flavonoids derived from *Chrysanthemum* species have been reported to possess anti‐inflammatory effects, as they help suppress nitric oxide production and inflammatory cytokine levels in LPS‐stimulated RAW264.7 cells [33]. These results suggest that DO alleviates LPS‐induced airway inflammation by downregulating inflammatory cytokines, with linarin, a major component of DO, playing a key role in its anti‐inflammatory activity.

In ALI, LPS causes airway inflammation in ALI through toll‐like receptor 4‐mediated NF‐κB signaling, resulting in the overproduction of cytokines [18, 19]. NF‐κB activation regulates pro‐inflammatory cytokines release and recruits the NLRP3 inflammasome complex [36]. This process activates caspase‐1, which converts IL‐1β and IL‐18 into their active forms, intensifying inflammation [36, 37]. Additionally, TXNIP contributes to the priming of NLRP3 inflammasome activation, enhancing pro‐inflammatory cytokines release in the ALI mouse model [10, 11, 37]. In our study, DO treatment reduced p65NF‐κB phosphorylation and inhibited TXNIP upregulation. Additionally, DO suppressed NLRP3 inflammasome activation by downregulating caspase‐1 and IL‐1β. In the subsequent study, linarin effectively inhibits p65NF‐κB phosphorylation and TXNIP upregulation in TNF‐α‐stimulated NCI‐H292 cells. Herbal medicines with anti‐inflammatory effects have been shown to significantly reduce airway inflammation by inhibiting p65NF‐κB and TXNIP/NLRP3 inflammasome in the ALI mouse model [11]. These findings indicate that linarin's ability to suppress p65NF‐κB and TXNIP/NLRP3 inflammasome activation plays a key role in mitigating airway inflammation, likely contributing to the overall anti‐inflammatory effects of DO in the LPS‐induced ALI model.

The accumulation of ROS triggered by LPS worsens inflammation in ALI by promoting the release of pro‐inflammatory cytokines and enhancing inflammatory cell infiltration [38, 39, 40]. Nrf‐2 plays a crucial role in mitigating LPS‐induced oxidative stress in ALI by regulating the expression of essential antioxidant enzymes such as HO‐1 and NQO‐1 [3, 41]. HO‐1 and NQO‐1 mitigate oxidative stress damage through carbon monoxide and biliverdin generation during heme degradation, a process that occurs in LPS‐induced ALI [41, 42]. NQO‐1 can detoxify reactive quinones and act as a reductase of coenzyme Q_10_, superoxide, and vitamin E in response to oxidative stress [43]. In our research, DO and its bioactive compound linarin significantly enhanced Nrf‐2 nuclear translocation and upregulated HO‐1, GSH, and NQO‐1 levels, effectively reducing ROS generation and lipid peroxidation in TNF‐α‐stimulated NCI‐H292 cells. In the LPS‐induced ALI model, DO promoted Nrf‐2 nuclear translocation and increased the levels of HO‐1, GSH, and NQO‐1 while effectively reducing ROS accumulation and lipid peroxidation. Previous studies have shown that the Nrf‐2/NQO‐1/HO‐1 signaling pathway contributes to protection against oxidative stress‐related damage in the LPS‐induced ALI model [20, 23]. Our findings suggest that the activation of the Nrf‐2/NQO‐1/HO‐1 pathway and the antioxidant properties of linarin, the primary active component of DO, are believed to be closely linked to DO's protective effects against LPS‐induced oxidative stress and lung injury.

*Dendranthema oreastrum* is a perennial herb of the Compositae family, has been traditionally used in Korean medicine to treat respiratory and inflammatory conditions [33]. Its anti‐inflammatory effects are linked to the suppression of pro‐inflammatory mediators in LPS‐treated RAW264.7 cells. *D. oreastrum* contains luteolin, apigenin, acacetin, and quercetin [27]. Linarin, the major component of methanolic extracts from *D. oreastrum var*. downregulates the mRNA levels of TNF‐α, IL‐6, and IL‐1β via suppression of p65NF‐κB in LPS‐stimulated human macrophage cells [43]. Additionally, it reduces NO production and inflammatory cytokines by inhibiting the MAPKs and NF‐κB pathways in LPS‐stimulated RAW264.7 cells [44, 45]. The antioxidant activity of linarin was evidenced by a decrease in TBARS levels and an increase in GSH content in embryonic rat cardiomyocyte H9C2 cells [46]. Previous studies have demonstrated that linarin prevents dry eye disease and neuropathy by inhibiting inflammation through the TXNIP/NLRP3 inflammasome pathway [47, 48]. However, the effects of *D. oreastrum* on an ALI model, as well as its underlying anti‐inflammatory or anti‐oxidant mechanisms, have not yet been explored. Based on previous studies on the effects of linarin, our research findings indicate that DO significantly attenuated airway inflammation and oxidative stress by inhibiting p65NF‐κB and TXNIP/NLRP3 inflammasomes while activating the Nrf‐2/NQO‐1/HO‐1 signaling in an LPS‐induced ALI mouse model and TNF‐α‐stimulated NCI‐H292 cells. In our study, DO exhibited protective effects in the LPS‐induced mouse model. Although these findings have notable strengths, certain limitations exist. We further confirmed the inhibitory activity of the TXNIP/NLRP3 inflammasome and the antioxidant activity of linarin, the major compound of DO, in NCI‐H292 cells. Thus, the anti‐inflammatory and anti‐oxidant effects of linarin may play a major role in the protective effects of DO in airway inflammation of the LPS‐induced ALI model by inhibiting the TXNIP/NLRP3 inflammasome and activating the Nrf‐2 pathways. However, the anti‐inflammatory and anti‐oxidant mechanisms of the major compounds in DO have not yet been fully elucidated in the ALI model. Therefore, further studies are needed to investigate its therapeutic potential and evaluate linarin as a possible treatment for the ALI model.

## 5 Conclusions

This study is the first to reveal that DO treatment alleviates airway inflammation and oxidative stress in both an LPS‐induced ALI mouse model and TNF‐α‐stimulated NCI‐H292 cells. These protective effects are linked to the suppression of the p65NF‐κB and TXNIP/NLRP3 inflammasome pathways, along with the activation of the Nrf‐2/NQO‐1/HO‐1 signaling pathway. Additionally, linarin's protective role is linked to the suppression of the p65NF‐κB and TXNIP/NLRP3 inflammasome pathways, as well as the modulation of the Nrf‐2/NQO‐1/HO‐1 signaling pathway in TNF‐α‐stimulated NCI‐H292 cells. These findings indicate that the methanolic extract of DO and its key active compound linarin may have therapeutic potential in reducing airway inflammation and oxidative stress, potentially aiding in ALI treatment.

## Author Contributions

Conceptualization: In‐Chul Lee and Young‐Bae Ryu. Methodology: Ji‐Hye Ha, Seong‐Hun Jeong, Ju‐Hong Kim, and In‐Chul Lee. Formal analysis: Seong‐Hun Jeong, Ji‐Hye Ha, and Ba‐Wool Lee. Investigation: Ji‐Hye Ha, Da‐Hye Yi, and Ba‐Wool Lee. Resources: In‐Chul Lee. Data curation: Ju Hwan Jeong, Ji‐Young Park, Hyung Jae Jeong, and Hyun‐Jae Jang. Writing – original draft preparation: Ji‐Hye Ha and Ba‐Wool Lee. Writing – review and editing: In‐Chul Lee and Hyung‐Jun Kwon. Visualization: Ji‐Hye Ha, Ju‐Hong Kim, and Ba‐Wool Lee. Supervision: Young‐Bae Ryu and In‐Chul Lee. Project administration: Hyung‐Jun Kwon and In‐Chul Lee. Funding acquisition: In‐Chul Lee. All authors have read and agreed to the published version of the manuscript.

## Conflicts of Interest

The authors declare no conflicts of interest.

## Supporting information


**Data S1.** Supporting Information.

## Data Availability

The data that support the findings of this study are available from the corresponding author upon reasonable request.
