# Supplementary material for: Dendranthema oreastrum (Hance) Y.Ling Attenuates Oxidative Stress and Airway Inflammation in a Murine Model of Lipopolysaccharide‐Induced Acute Lung Injury
Source: Environ Toxicol. 2025 Apr 23;40(10):1220–32. doi: 10.1002/tox.24520 (PMC12432812; doi:10.1002/tox.24520)
Supplement: Supplementary file 1 — Data S1. Supporting Information. [file TOX-40-1220-s001.docx]

**Supplement Figure 1**

**
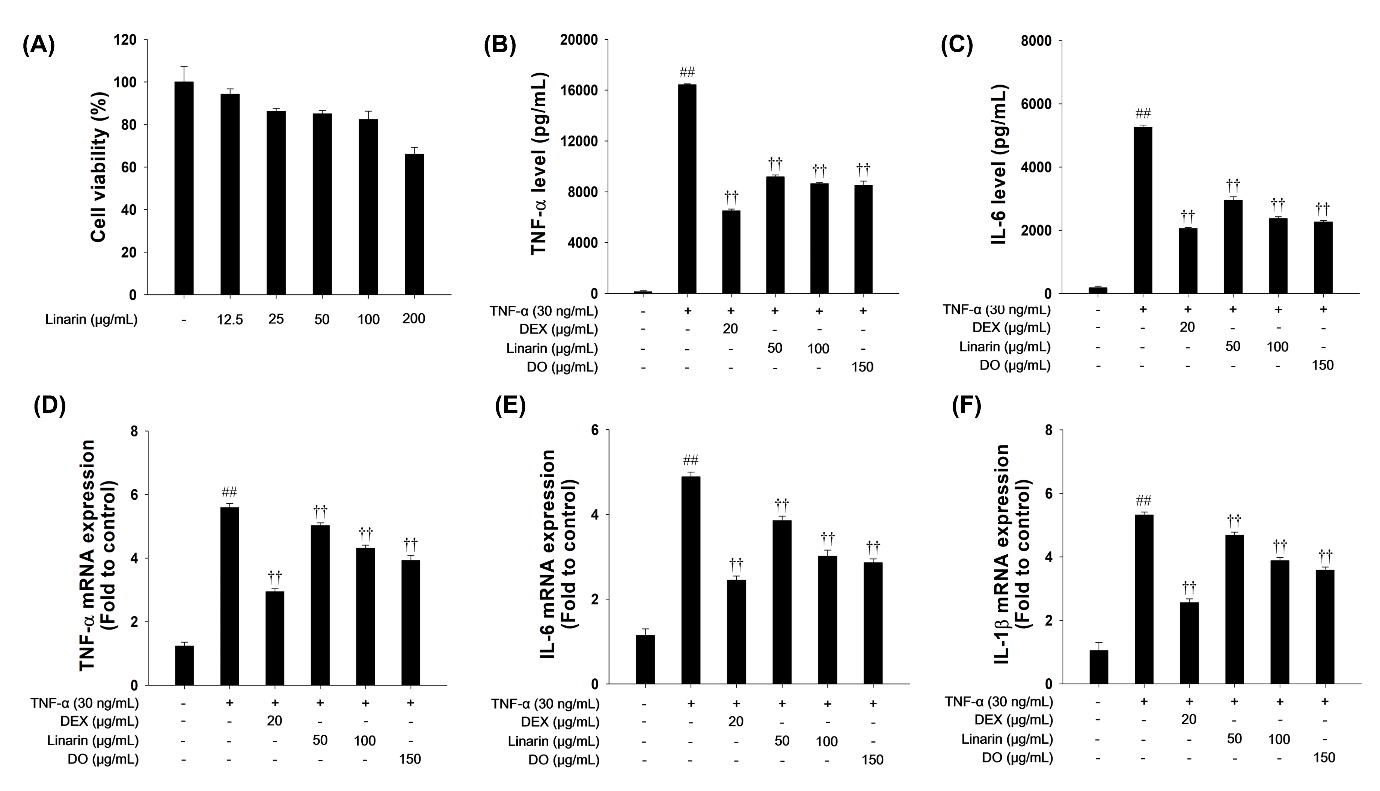
**

**Supplement Figure 2**

**
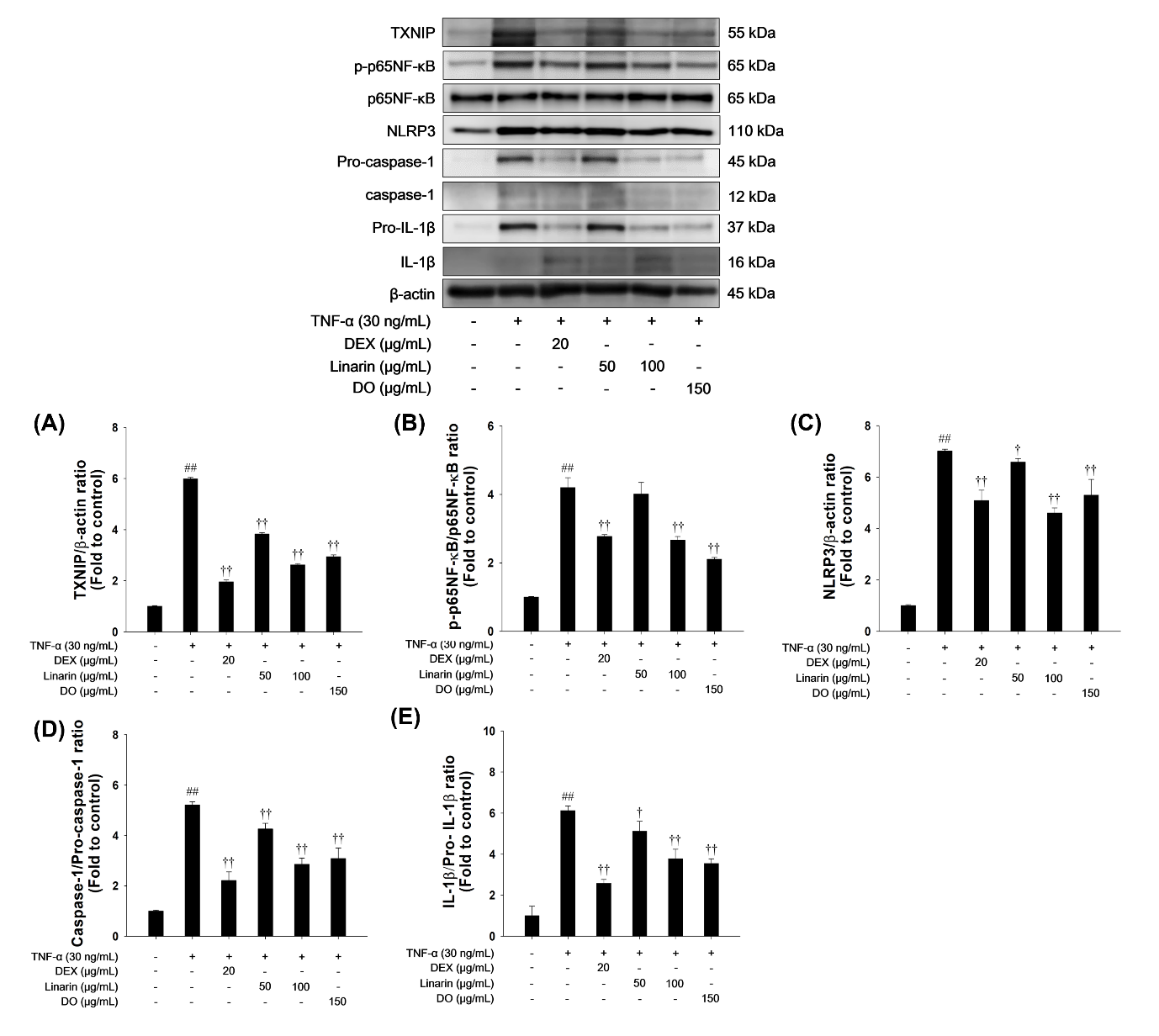
**

**Supplement Figure 3**

**
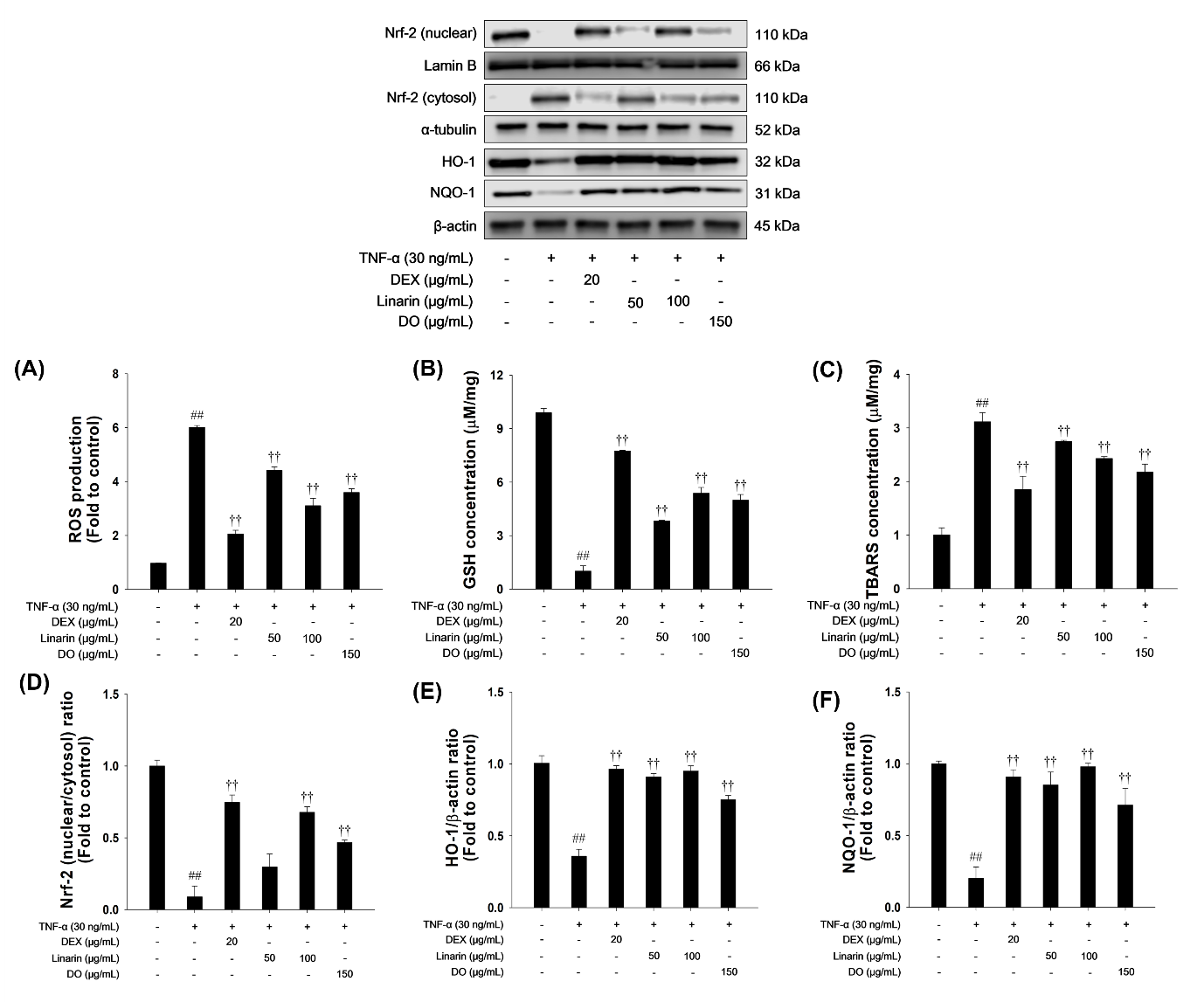
**

**Supplement Figure legends**

**Supplement Figure 1. Effects of linarin on NCI-H292 cell viability and pro-inflammatory cytokine.**

**(A)** Effects of linarin concentration on NCI-H292 cell viability, **(B)** TNF-α and **(C)** IL-6 were determined by ELISA. The levels of **(D)** TNF-α, **(E)** IL-6, and **(F)** IL-1β were determined by RCR. The values are expressed as the means ± SD (*n* = 3). Significance: ^##^ *p* < 0.01 vs control; ^††^ *p* < 0.01 vs TNF-α-stimulated cells, respectively.

**Supplement Figure 2. Effects of linarin on p65NF-κB and TXNIP/NLRP3 inflammasome pathway in NCI-H292 cell.**

**(A)** TXNIP, **(B)** p65NF-κB, **(C)** NLRP3, **(D)** caspase-1, and **(E)** IL-1β. The values are expressed as the means ± SD (*n* = 3). Significance: ^##^ *p* < 0.01 vs control; ^†, ††^ *p* < 0.05 and < 0.01 vs TNF-α-stimulated cells, respectively.

**Supplement Figure 3. Effects of linarin on Nrf-2 pathway and oxidative stress in NCI-H292 cell.**

**(A)** ROS production, **(B)** GSH, and **(C)** TBARS. The expression of **(D)** Nrf-2, **(E)** HO-1, and **(F)** NQO-1. The values are expressed as the means ± SD (*n* = 3). Significance: ^##^ *p* < 0.01 vs control; ^†, ††^ *p* < 0.05 and < 0.01 vs TNF-α-stimulated cells, respectively.
